# miR-375 Suppresses IGF1R Expression and Contributes to Inhibition of Cell Progression in Laryngeal Squamous Cell Carcinoma

**DOI:** 10.1155/2014/374598

**Published:** 2014-08-12

**Authors:** Jie Luo, Jianhui Wu, Zenghong Li, Hao Qin, Bin Wang, Thian-Sze Wong, Weiqiang Yang, Qing-Ling Fu, Wenbin Lei

**Affiliations:** ^1^Department of Otorhinolaryngology, The First Affiliated Hospital of Sun Yat-sen University, Otorhinolaryngology Institute, Sun Yat-sen University, Guangzhou, China; ^2^Department of Surgery, Queen Mary Hospital, The University of Hong Kong, Hong Kong; ^3^Department of Otorhinolaryngology, The First People's Hospital of Foshan, Foshan, China; ^4^Otorhinolaryngology Hospital, The First Affiliated Hospital, Sun Yat-sen University, 58 Zhongshan Road II, Guangzhou, Guangdong 510080, China

## Abstract

MicroRNAs (miRNAs) are small noncoding RNA molecules which are involved in tumorigenesis and development. To investigate their role in primary laryngeal squamous cell carcinoma (LSCC), miRNA GeneChips were used to screen the differentially expressed miRNA, and then validated by real-time quantitative PCR in LSCC samples, we found that miR-375 was frequently downregulated in primary LSCC tissues. The tumor-suppressive effect of miR-375 was determined by in vitro assays; through gain-of-function studies we demonstrated that miR-375 can inhibit LSCC cell (SNU-48 and SNU-899) proliferation, motility, and invasion, and promote their apoptosis. In addition, bioinformatics tools TargetScan, PicTar, and Miranda were used to investigate the potential target of miR-375; bioinformatics analysis and dual-luciferase reporter assay indicated that IGF1R was a novel direct target of miR-375. Ectopic transfection of miR-375 led to a significant reduction in IGF1R and its downstream signaling molecule AKT at both the mRNA and protein levels in LSCC cells. Our results suggested that downregulation of miR-375 is one of the molecular mechanisms for the development and progression of LSCC by directly targeting IGF1R and affecting its downstream AKT signaling pathways. Furthermore, miR-375 and IGF1R may serve as a novel therapeutic target for LSCC.

## 1. Introduction

Laryngeal carcinoma is one of the most common malignant neoplasms. With an estimated incidence rate of 5.1/100,000 in males worldwide in 2008, it heavily threatens their health and quality of life [1, 2]. Squamous cell carcinoma is the predominant histological type accounting for over 95% of laryngeal carcinoma. There have been reported changes in the expression of many oncogenes (Ras [3], ZFX [4], and Aurora-A [5]) and tumor suppressor genes (BMI1 [6], TSLC1 [3, 7], and p-AKT [8]) in LSCC. These changes could affect cancer development by modulating downstream signal transduction pathways such as the well-known AKT signaling pathway [9, 10]. Therefore, a deeper understanding of these molecular mechanisms will help us find new diagnostic and therapeutic approaches to this disease and improve the prognosis of LSCC patients.

MicroRNA are a class of small noncoding RNAs (20–25 ribonucleotides) that play an important role in regulating gene function. Upon binding to the 3′-untranslated region (UTR) of their target messenger RNAs, the expression of their target gene is repressed or stopped by multiple mechanisms including enhanced translational repression and mRNA degradation [11, 12]. Since the relationship between miRNAs and cancer has been first elucidated in a study of B cell chronic lymphocytic leukaemia [13], an increasing number of studies have shown that the biological functions of miRNAs are highly correlated with human carcinogenesis of lung, breast, ovary, and liver, and laryngeal carcinoma is not an exception [14]. These studies suggest a critical role of miRNAs in tumorigenesis and development [15]. Previous studies have shown several dysregulated miRNAs in LSCC through expressing array profiling. The target genes of these miRNAs and the related cancer pathways have been further identified. For example, miR-1 was downregulated in LSCC tissues and suppressed the invasion and migration by targeting FN1 in LSCC cell [16], and miRNA-1297 was originally found to regulate cell proliferation and differentiation in LSCC by targeting PTEN [17]. However, further understanding of the molecular mechanisms of miRNA in LSCC is needed before providing better therapeutic approach for LSCC patients. In present study, we aimed at identifying the most aberrantly expressed miRNA in LSCC tissues, investigating the biological functions of this miRNA in LSCC, and further discussing the underlying mechanisms.

## 2. Materials and Methods

### 2.1. Clinical Samples

We obtained paired larynx squamous cell carcinoma (LSCC) and their corresponding nontumor tissues (located more than 10 mm from the tumors) from 40 patients who underwent primary surgical resection of LSCC between March 2012 and September 2013 in our department. All samples were confirmed by pathology. Samples were snap-frozen in liquid nitrogen after resection and stored at −80°C. Patients were excluded if they had recurrent LSCC or had primary LSCC but underwent chemoradiotherapy before surgical operation. This study was approved by the Human Research Ethics Committee of Sun Yat-sen University (the ethical number: [2013  23]).

### 2.2. Gene Expression Microarray

Total RNA was extracted from LSCC tumor and corresponding nontumor samples using the mirVana miRNA isolation kit (Ambion). Before microarray (Affymetrix) analysis, RNA quality was confirmed by RNA integrity number using Agilent 2100 bioanalyzer (Agilent Technologies) at the University of Hong Kong. All samples had an RNA integrity number greater than 7.0.

### 2.3. Cell Culture and Transfection

Two LSCC cell lines (SNU899 and SNU46) were kindly provided by Professor Thian-Sze Wong (University of Hong Kong). Cells were maintained in RPMI-1640 (Hyclone) containing 10% fetal bovine serum (FBS, Hyclone), 100 units/mL penicillin, and 100 *μ*g/mL streptomycin, in a humidified incubator of 5% CO_2_ at 37°C. The hsa-miR-375 molecule (miR-375 mimics) and RNA-oligonucleotides negative control (miR-NC) were obtained from GenePharma corporation. Transfection of cells was performed using Oligofectamine (Invitrogen, Carlsbad, CA) according to the manufacturer's protocol. Culture medium was changed 24 h after transfection. Transfection efficiency was evaluated by real-time PCR from three experiments.

### 2.4. Extraction of Total RNA

Total RNA was isolated from 40 pairs of frozen tissue samples using the mirVana miRNA Isolation kit (Ambion) according to the manufacturer's protocol. For human LSCC cells SNU46 and SNU899, total RNA was extracted using TRIzol (Invitrogen, Carlsbad, California, USA).

### 2.5. Quantitative Reverse Transcription PCR

Quantitative PCR was carried out in triplicate with primers for miR-375, U6, IGF1R, and GAPDH using 7900 HT real-time PCR system (Applied Biosystems) following standard quantitative PCR protocol. The primers used were as follows: miR-375: (F) 5′-CAGGGTCCGAGGTATT-3′ and (R) 5′-CTGCTTTGTTCGTTCG-3′; U6: (F) 5′-CTCGCTTCGGCAGCACA-3′ and (R) 5′-AACGCTTCACGAATTTGCGT-3′; IGF1R: (F) 5′-AACCCCAAGACTGAGGTGTG-3′ and (R) 5′-TGACATCTCTCCGCTTCCTT-3′; GAPDH: (F) 5′-CCACCCATGGCAAATTCCATGGCA-3′ and (R) 5′-TCTAGACGGCAGGTCAGGTCCACC-3′.

The expression level was normalized against endogenous U6 and GAPDH for miR-375 and IGF1R, respectively. qRT-PCR results were analyzed and expressed as relative gene expression, which were then converted to fold changes by 2^−ΔΔCt^ method.

### 2.6. Proliferation Assay

LSCC cells (10^5^/well) transfected with miR-375 mimics or miR-NC were seeded into 96-well plate and cultured for 24, 48, 72, and 96 h. For MTT assay, 3-(4,5-dimethylthiazol-2-yl)-2,5-diphenyltetrazolium bromide (MTT) (0.5 mg/mL, pH 4.7; Sigma) was added 4 h before the end of the culture time. At indicated times, the supernatant was removed, 150 *μ*L dimethyl sulfoxide (DMSO) was added, and the plate was shaken for 15 minutes at room temperature. The absorbance at 490 nm was measured using a microplate reader (Bio-Rad, Hercules, CA, USA). The mean values from 5 identical wells were used to construct the cell growth value.

### 2.7. Wound Healing Assay

Transfected cells (10^5^/well) were cultured in six-well plates for 24 h and then 5 wounds were made on the cell monolayer using a 20 *μ*L pipette tip. Cells were washed three times with PBS and then cultured in RPMI-1640. Cells were imaged at 0, 6, 12, and 24 h after the wounding. The distance migrated by miR-375 mimics-treated cells relative to that migrated by miR-NC-treated cells was determined.

### 2.8. Invasion Assay

Cell invasion was determined using transwell invasion chambers (corning). 2 × 10^4^ cells were seeded on the upper side of the polycarbonate transwell filter with Matrigel (BD Biosciences) and cultured in 200 *μ*L RPMI-1640. The lower chamber was filled with 500 *μ*L RPMI-1640 with 10% FBS to attract cells. After cells were incubated for 36 hours at 37°C and 5% CO_2_, those left on the upper chamber were removed with a cotton swab. The filter was then fixed with 95% ethanol for 20 min. Cells on the bottom of the filter were stained with 4 g/L crystal violet, photographed in five independent 20x magnification fields, and counted.

### 2.9. Apoptosis Assay

The apoptosis ratio was analyzed using the Annexin V-FITC Apoptosis Detection Kit (KeyGEN Biotech, China) according to the manufacturer's instructions. Cells (2 × 10^5^) were transfected with miR-375 mimics or miR-NC for 48 h and then harvested. Cells were resuspended in 300 *μ*L binding buffer containing Annexin V-FITC and PI and incubated at room temperature in the dark for 15 min. Stained cells were analyzed by flow cytometry (FACScan; BD Biosciences) and the percentage of apoptotic cells was obtained. Samples from three independent experiments were used for analysis.

### 2.10. Dual-Luciferase Reporter Assay

A 1572 bp fragment from the 3′-UTR of the IGF1R gene (containing two potential miR-375-targeting sites at position 2276 and 2993) was cloned into the hRlu site of luciferase reporter vector PsiCHECK^UM^ (Promega, Madison, WI, USA) and the recombinant plasmid was designated PsiCHECK^UM^-wt. The site-directed mutagenesis of miR-375 target site in the 3′-UTR of the IGF1R gene was performed using the site-directed mutagenesis kit (TaKaRa, Dalian, China). We created 3 corresponding mutant constructs by mutating the seed regions of the miR-375 binding sites: PsiCHECK^UM^-mut1 plasmid contains mutated binding site at position 2276–2282, PsiCHECK^UM^-mut2 plasmid contains mutated binding site at position 2993–2999, and PsiCHECK^UM^-mut3 plasmid contains mutated binding sites at both positions as mentioned above. The constructs were verified by sequencing.

SNU-46 cells (2 × 10^4^/well) were seeded into 24-well plates and cotransfected with 0.5 *μ*g of the luciferase reporter gene construct and 1 *μ*L miRNA mimic or inhibitor (50 *μ*M) using Lipofectamine 2000 (Invitrogen). After 48 h, Firefly and Renilla luciferase signals were determined using the dual-luciferase reporter assay system (Promega) following the manufacturer's instructions. The pRL-TK vector (Promega) was cotransfected as an internal control for the normalization of the transfection efficiency. Activity of Renilla luciferase was normalized to Firefly luciferase. Samples were assayed in triplicate.

### 2.11. Western Blot Analysis

Cells were collected in lysis buffer, and protein was extracted and analyzed by western blotting as described previously [18]. The antibodies used include anti-IGF1R antibody (1 : 500; Abcam) and anti-phosphorylated Akt antibody (Ser473; 1 : 2000; Cell Signaling Technology).

### 2.12. Statistical Analysis

Statistical analyses were performed using GraphPad Prism 5 software. Wilcoxon signed-ranks test was used to analyze the differences in miRNA expression. Patients were categorized into groups with high (above median) or low (below median) miRNA375 expression. The association of miRNA375 expression with LSCC patients' clinicopathological features was analyzed by the *χ* ^2^ test. Differences were considered significant when the *P* value was less than 0.05.

## 3. Results

### 3.1. miRNA Profiling in Human LSCC

To investigate the role of miRNAs in LSCC, MicroRNA Expression GeneChips were used for expression profiling analysis. Differential expression between LSCC tissues and their corresponding nontumor tissues was defined using a cutoff value of 5-fold change (Tables 1 and 2). Among the miRNAs, miR-375 was found with the largest fold change in expression array and thus was chosen for further study.

### 3.2. miR-375 Was Downregulated in LSCC

**Figure 1:**
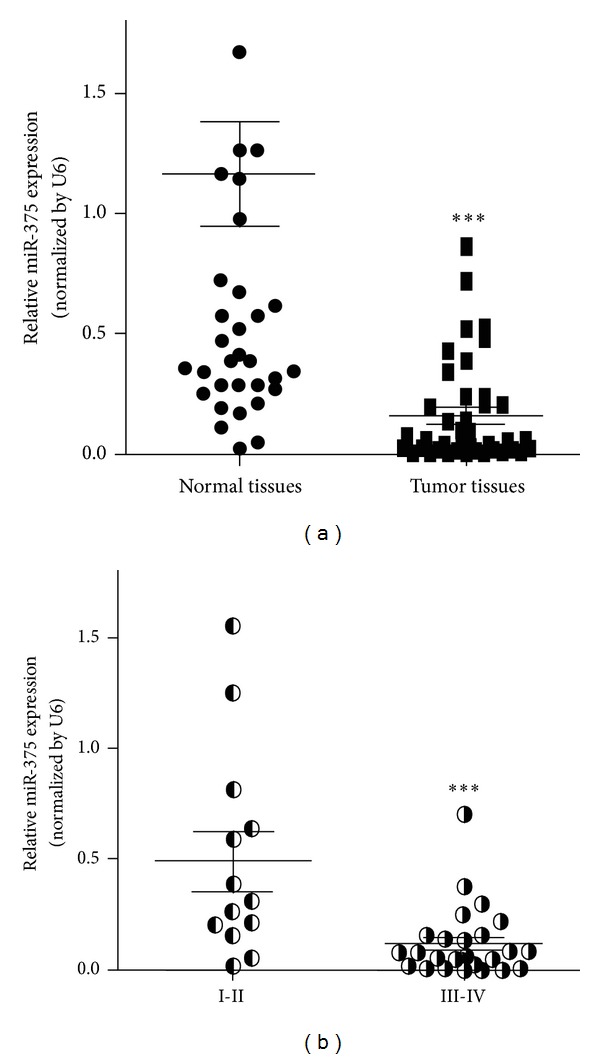
miR-375 was downregulated in LSCC tissue samples. (a) Expression levels of miR-375 in normal LSCC tissues and paired normal tissues were analyzed by qRT-PCR and normalized to the levels of U6. (b) Relative expression levels of miR-375 in different stages of cancer tissues. Forty pairs of samples were divided into two groups according to the patient's clinic stage. (∗∗∗) Significant difference when compared with normal LSCC tissues (*P* < 0.001).

To further assess the expression of miR-375, qRT-PCR was conducted on 40 pairs of LSCC tissues. miR-375 expression was notably lower in cancer tissues compared with their paired paracarcinoma tissues, similar to the previous miRNA expression array data. These results indicate that miR-375 expression is reduced in human LSCC tissues (Figure 1(a)).

### 3.3. Relationship between miR-375 Expression and Tumor Clinicopathologic Features

All patients were staged according to the 2002 Union Form International Cancer Control (UICC) staging classification system for laryngeal cancer. The levels of miR-375 expression in stage III-IV cases were much lower than those in stage I-II cases, suggesting that miR-375 expression correlated with LSCC malignancy (Figure 1(b)). We did not find any significant association between the expression of miR-375 and patient age, gender, nodal status, pathological grade, and tobacco and alcohol use. Only pT status (*P* = 0.035) and UICC clinical stage (*P* = 0.041) were linked to miR-375 expression (Table 3).

### 3.4. miR-375 Suppressed LSCC Cell Proliferation

**Figure 2:**
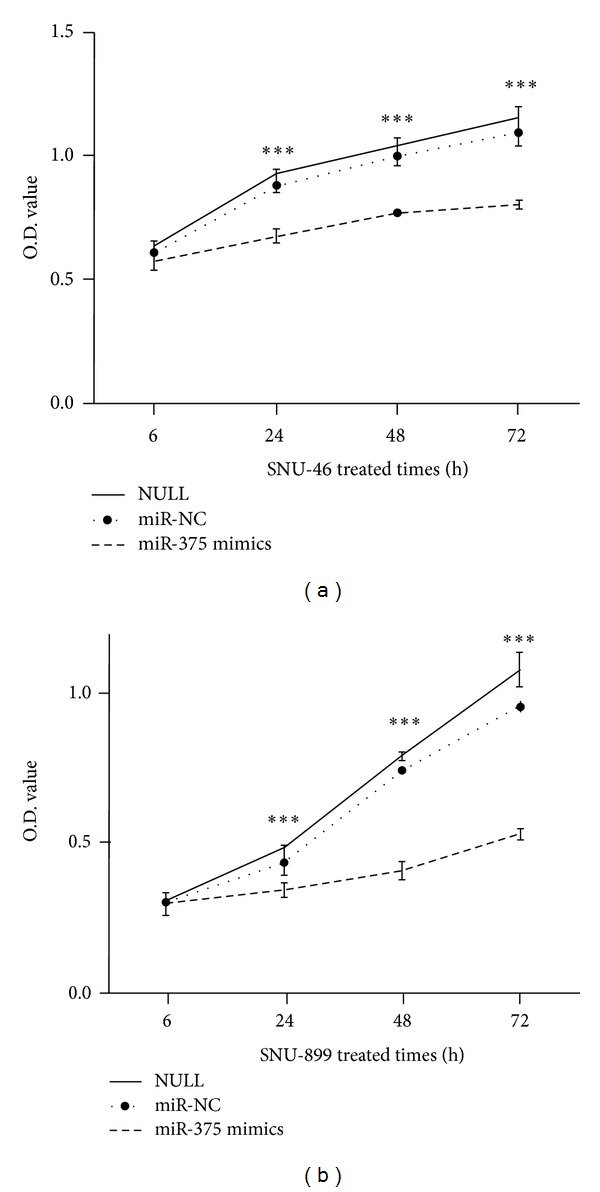
miR-375 overexpression inhibited LSCC cell proliferation. (a) Overexpression of miR-375 decreased SNU-46 cell growth. (b) Overexpression of miR-375 decreased SNU-899 cell growth. (∗∗∗) Significant difference when compared with the negative-control (miR-NC) and blank-control (NULL) group (*P* < 0.001).

miR-375 was found to be downregulated in LSCC tissues by qRT-PCR, implicating its potential role in regulating LSCC cells' biological behaviors. To further determine whether miR-375 affects the proliferation of LSCC cells, miR-375 mimics or miR-NC were transfected into LSCC cell lines and MTT assay was performed. Satisfactory transfection efficiency was observed in these cells, and overexpression of miR-375 significantly suppressed the proliferation of SNU-46 and SNU-899 cells at 24, 48, 72, and 96 h after transfection, respectively (Figure 2, *P* < 0.05).

### 3.5. miR-375 Promoted Apoptosis in LSCC Cells

**Figure 3:**
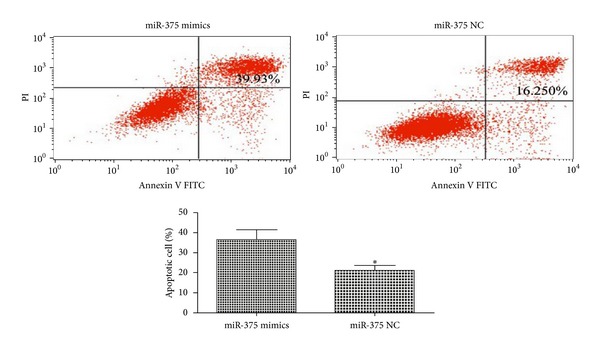
Apoptosis assay showing induction of apoptosis after miRNA-375 overexpressed by miRNA-375 mimics in SNU46 cells. Early and late apoptotic cells were combined as annexin V-positive cells that were employed as the criterion to calculate the percentage of cell apoptosis. (∗) Significant difference when compared with the miR-NC group (*P* < 0.05).

To evaluate the effect of miR-375 on LSCC cell apoptosis, FACS analysis for apoptosis was performed using Annexin-V-FITC and PI dye. The percentage of apoptotic cells was significantly increased in response to miR-375 overexpression compared with miR-NC overexpression in SNU46 cells. In SNU899 cells, an increase in apoptotic cells was observed with miR-375 overexpression compared with miR-NC overexpression. These results indicated the antiapoptotic role of miR-375 in LSCC cells (Figure 3).

### 3.6. miR-375 Inhibited LSCC Cell Migration and Invasion

**Figure 4:**
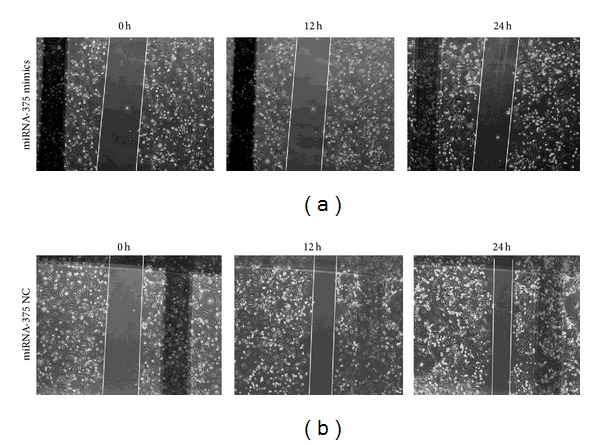
miR-375 suppressed LSCC cell migration. Wound healing assay shows that SNU-46 cell motility could be effectively suppressed by miR-375 mimics (a) and miR-NC (b). Representative images were taken at 0, 12, and 24 h after scratching.

**Figure 5:**
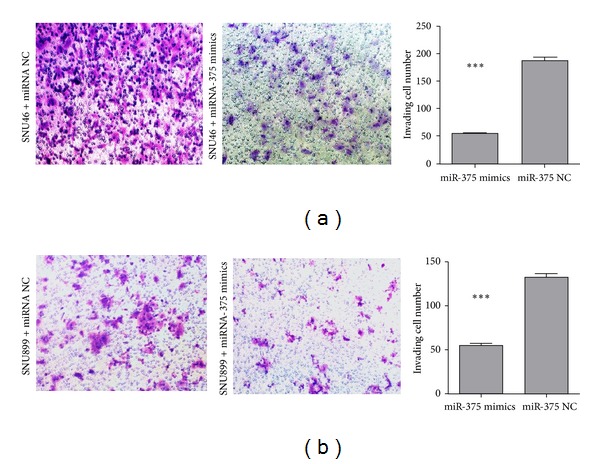
Transwell invasion assay. (a) Upregulation of miR-375 in SNU-46 cells significantly inhibited invasion compared with the control group. (b) Invasion of SNU-899 cells was inhibited when cells were transfected with miR-375 mimics compared with miR-NC. Cells on the bottom of the invasion chamber were fixed, stained, and photographed, and the number of cells was counted. The results are expressed as mean ± SD of three independent experiments. (∗) Significant difference when compared with the miR-NC group (*P* < 0.05).

To investigate whether miR-375 overexpression also significantly reduces the migration and invasion of LSCC cells, wound healing and transwell invasion assays were conducted. We found that overexpression of miR-375 led to a decrease in SNU-46 cell migration (Figure 4) and significantly inhibited invasion of LSCC cells (Figure 5). These results suggested that miR-375 contributes to suppress both LSCC cell migration and invasive capacity in vitro.

### 3.7. IGF1R Was a Potential Target of miR-375

**Figure 6:**
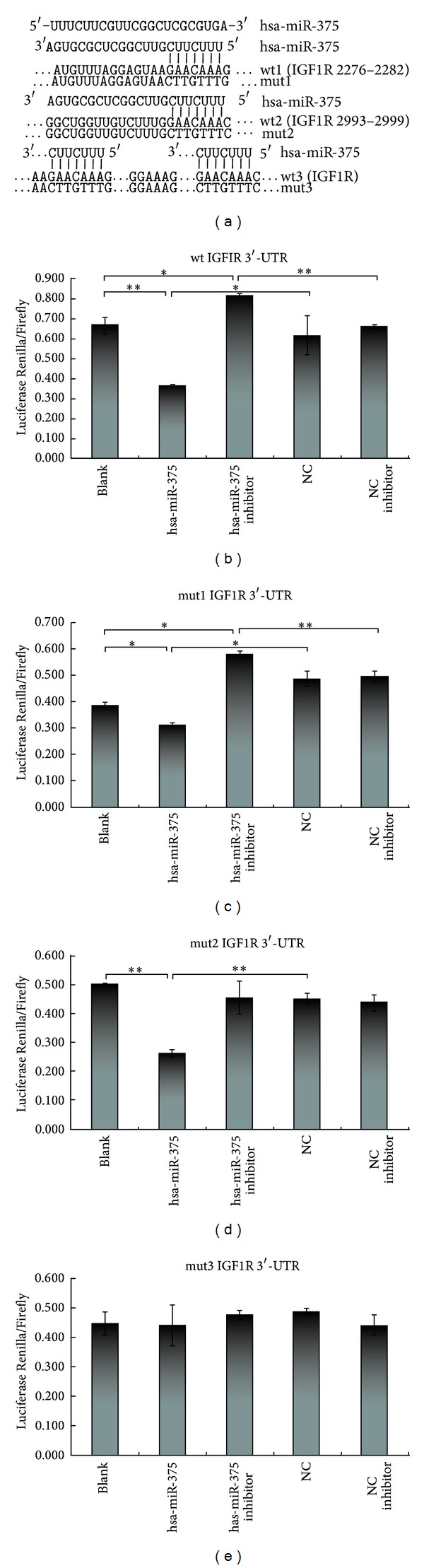
miR-375 inhibited the expression of IGF1R by targeting its 3′-UTR. (a) Schematic diagram showing the two potential targeting sites of miR-375 in the 3′-UTR of IGF1R and gene sequence included in four luciferase reporter plasmids, respectively. (b–e) Relative luciferase activities of IGF1R-wt and IGF1R-mut reporters were obtained by cotransfection of scrambled control miRNA, miR-375 mimic or miR-375 inhibitor, and psiCHECK-2-IGF1R-3′UTR reporter plasmid and calculated as the ratio of Firefly/Renilla activities and normalized to those of the control. (∗) Significant difference when compared with the control (*P* < 0.05). (∗∗) Significant difference when compared with the control (*P* < 0.01).

**Figure 7:**
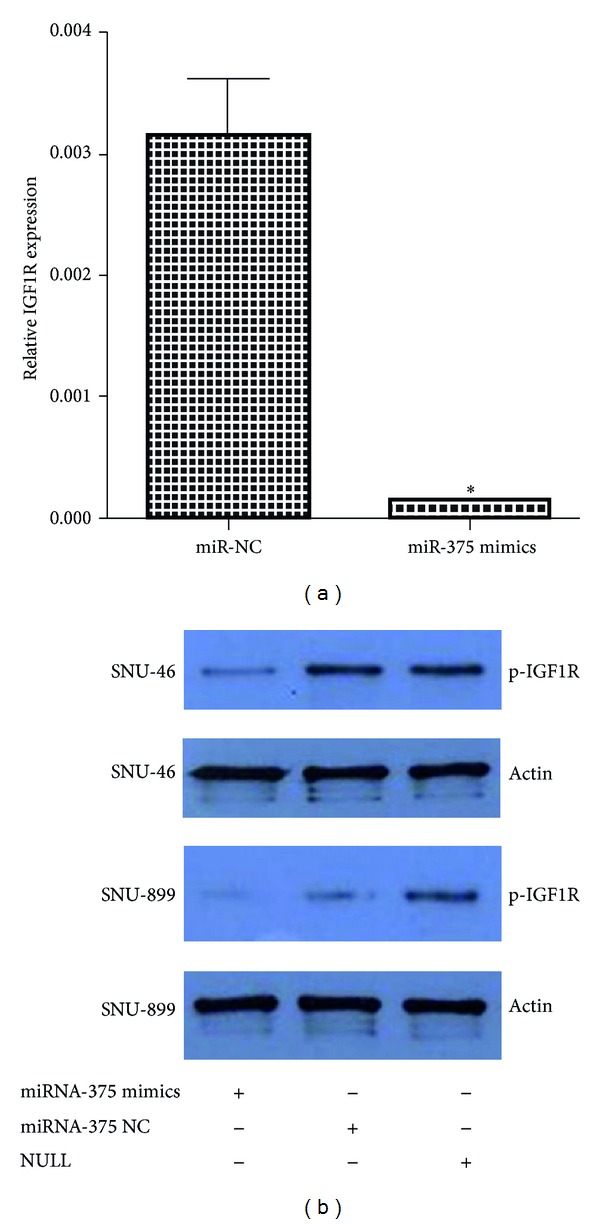
miR-375 inhibited IGF1R expression at both mRNA and protein levels. SNU-46 cells transfected with miR-NC and mi-R375 were analyzed by RT-PCR (a). Overexpression of miR-375 inhibited IGF1R expression in SNU-46 and SNU-899 cells at the protein level (b). (∗) Significant difference when compared with the control (*P* < 0.05).

**Figure 8:**
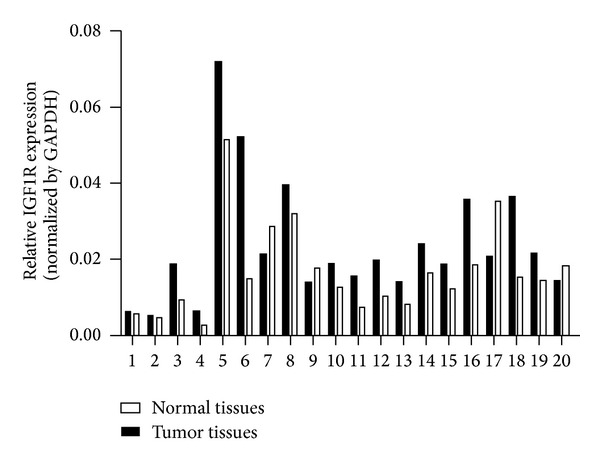
IGF1R was upregulated in LSCC tissue samples. Expression level of IGF1R was tested in 20 primary LSCC by qRT-PCR. Results showed that IGF1R was frequently upregulated in tumour tissues compared with their normal counterparts. GAPDH was used as an internal control.

**Figure 9:**
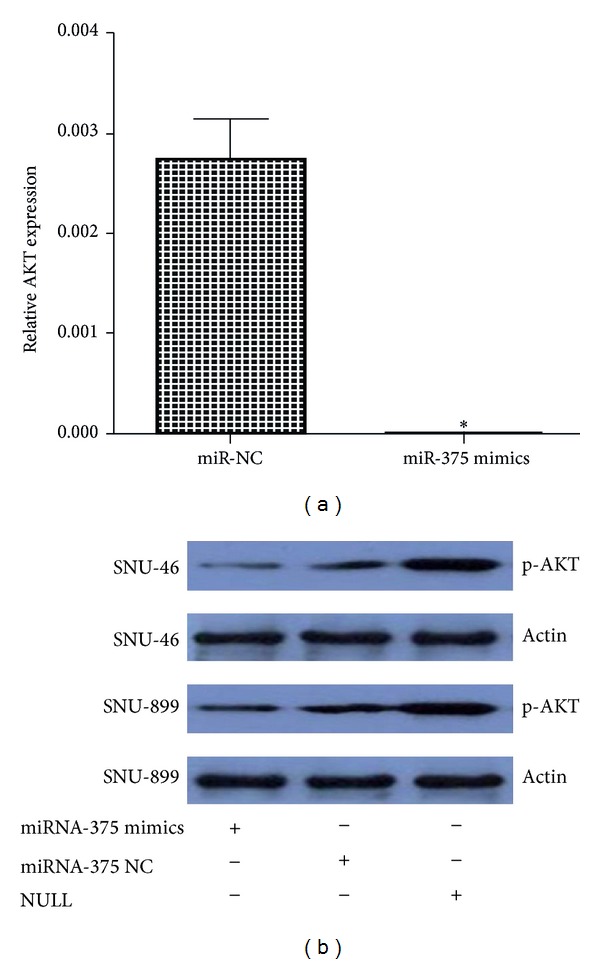
miR-375 overexpression regulated IGF1R signaling. AKT mRNA levels were determined by qRT-PCR and normalized to those of GAPDH. (∗) Significant difference when compared with control (*P* < 0.05) (a). Levels of p-Akt and actin were detected by western blot analysis (b).

Given the observation that miR-375 played an important role in regulating LSCC cells biological properties, we next investigated the potential targets of miR-375 using bioinformatics tools TargetScan, PicTar, and Miranda. All of these programs predicted IGF1R as a target of miR-375. Two potential miR-375 targeting sites were identified at position 2276 and 2993, respectively, in IGF1R 3′-UTR (Figure 6(a)). To confirm the target relationship, four luciferase reporter plasmids were generated, including the psiCHECK-2-wild-IGF1R-3′UTR reporter plasmid (wt) with the wild-type IGF1R target sequence and the psiCHECK-2-mutant-IGF1R-3′UTR reporter plasmids (mut1, mut2, and mut3) in which the conserved target sequence at position 2276 and/or 2993 was mutated. Plasmids mut1, mut2, and mut3 had the mutation at positions 2276, 2993, and both 2276 and 2993, respectively. The reporter plasmids were cotransfected with miR-375 mimic or miR-375 inhibitor into SNU-46 cells. miR-375 significantly inhibited the luciferase activity of wild-type reporter (wt) plasmid (Figure 6(b)) and partially inhibited that of the mutant reporter plasmids mut1 and mut2 (Figures 6(c) and 6(d)). In contrast, the luciferase activity was not affected by plasmid mut3 (Figure 6(e)), probably due to the mutation of both miR-375 binding sites in IGF1R 3′UTR. These data indicate that IGF1R was targeted directly by miR-375. We then transfected LSCC cell lines with miR-375 and miR-NC and confirmed the transfection efficiency using RT-PCR assays. Cells overexpressing miR-375 showed lower levels of IGF1R protein when compared with negative control cells (Figure 7). QRT-PCR was performed to investigate the expression of IGF1R in LSCC tissues; the result shows that this gene was significantly higher in tumor tissues than the adjacent normal tissues (Figure 8). Since IGF1R is a major player in the activation of the Akt signaling pathway, we also investigated the effect of miR-375 on the downregulation of Akt phosphorylation. As illustrated in Figure 9, although total Akt level was constant, the level of p-Akt was reduced after cells were treated with miR-375 mimic.

## 4. Discussion

In recent years, with the development of microarrays and PCR, many studies have suggested that dysregulation of miRNAs is closely related with tumor development [19–21]. However, these studies did not specify the mechanism of miRNAs in LSCC. Here we screened miRNAs from LSCC tissues and corresponding normal laryngeal epithelial tissues using microarrays and identified miR-375 as the miRNA with the most significant change in its expression between LSCC tissues and normal control tissues. We then analyzed 40 pairs of LSCC and normal surrounding tissues using qRT-PCR for verification and found that miR-375 expression was significantly lower in LSCC tissues and its level was closely related to patient's clinical stage.

These results suggest that miRNA-375 downregulation may be one of the molecular events for LSCC development. Originally found in pancreatic tissues, miR-375 is considered to be an evolutionarily conserved and islet-specific miRNA that functions as a regulator of insulin secretion [22]. Subsequent studies have shown that low expression of miR-375 was observed in many malignancies, such as liver cancer, esophageal cancer, and head and neck squamous cell carcinoma [14, 23–25]. Harris et al. have shown that miRNA-375 expression was significantly lower in the head and neck squamous cell carcinoma from miRNA expression profiling [26]. Similar results were also shown by others [25, 27]. miR-375 inhibits the expression of multiple target genes and is involved in almost all cell biological processes in cancers, including cell proliferation, apoptosis, differentiation, migration, and invasion. The change of miRNA-375 expression may be associated with cancer diagnosis and prognosis, and miR-375 could be a potential candidate target for cancer gene therapy [16, 25].

This study further investigated the role of miR-375 in regulating cell biological functions during the development of LSCC. We found that miR-375 significantly inhibited cell proliferation, migration, and invasion, while promoting apoptosis in two LSCC cell lines (SNU-46, SNU-899). Here we first proposed that miR-375 may be one of tumor suppressor genes in LSCC. To elucidate the molecular mechanism of LSCC development, we used bioinformatics to predict IGF1R as the target gene of miR-375. Although confirmed in squamous cell carcinoma of the esophagus [24], the relationship between miRNA and its target gene is affected by cell types and cell microenvironment; thus the same miRNA may play different roles in different cells [13]. We used dual-luciferase reporter assay to confirm the binding sites of miR-375 in the 3′UTR region in IGF1R mRNA in LSCC cell line SNU-46. qRT-PCR and western blotting were also used to confirm the effect of miR-375 in inhibiting IGF1R at both mRNA and protein levels. IGFIR is a member of the family of tyrosine kinase receptors and shows high level of expression in many tumor cells [28]. It is an important factor for cell malignant transformation [28–30]. Latest studies have suggested that overexpression of IGF1R in the blood of LSCC patients may serve as an independent indicator for tumor recurrence and poor prognosis [31]. IGF1R mediates a variety of important molecular signaling pathways via its adaptor protein IRS and plays a key role in regulating tumor development. Akt pathway is considered to be closely related to the development of head and neck squamous cell carcinoma [32, 33]. Interestingly, we found that not only the expression of IGF1R in LSCC tissues was significantly higher than the adjacent tissues, but also the levels of p-AKT mRNA and protein in LSCC cells with miR-375 overexpression were significantly decreased the same as the expression level of IGF1R. These results suggest that miR-375/IGF1R/AKT signaling pathway may play an important role in the development of LSCC. Further studies are required to confirm the mechanism.

In summary, this study has suggested that upregulation of IGF1R due to miR-375 depletion is an important molecular event in the development and progression of LSCC. miR-375 can significantly inhibit LSCC cell proliferation, migration, and invasion and promote apoptosis to suppress tumor via IGF1R-mediated AKT signaling pathway. Thus, miR-375 and IGF1R as the upstream molecules in AKT signaling pathway may serve as a novel therapeutic target for LSCC.

## Figures and Tables

**Table 1:** Downregulated miRNAs in LSCC tissues.

Downregulated miRNA	Sequence	Fold change
hsa-miR-375	UUUGUUCGUUCGGCUCGCGUGA	106.9796
hsa-miR-99a	AACCCGUAGAUCCGAUCUUGUG	13.27167
hsa-miR-30a	UGUAAACAUCCUCGACUGGAAG	11.03537
hsa-miR-574-3p	CACGCUCAUGCACACACCCACA	10.9491
hsa-miR-200b	UAAUACUGCCUGGUAAUGAUGA	9.396616
hsa-miR-148a	UCAGUGCACUACAGAACUUUGU	7.805731
hsa-miR-143	UGAGAUGAAGCACUGUAGCUC	6.750769
hsa-miR-141	UAACACUGUCUGGUAAAGAUGG	5.866387
hsa-let-7f	UGAGGUAGUAGAUUGUAUAGUU	5.637025
hsa-miR-200b∗	CAUCUUACUGGGCAGCAUUGGA	5.199535

∗When the two arms of precursor were added to produce mature miRNA separately, according to cloning experiments.

**Table 2:** Upregulated miRNAs in LSCC tissues.

Upregulated miRNA	Sequence	Fold change
hsa-miR-205	GUGAAAUGUUUAGGACCACUAG	77.25538
hsa-miR-25	CUCCCACUGCUUCACUUGACUA	33.99586
hsa-miR-193b∗	AACAUUCAUUGCUGUCGGUGGGU	33.9129
hsa-miR-342-5p	CUGUGCGUGUGACAGCGGCUGA	20.45821
hsa-miR-185	UAAGGUGCAUCUAGUGCAGAUAG	20.30513
hsa-miR-221	CACUAGAUUGUGAGCUCCUGGA	16.9373
hsa-miR-720	UGCGGGGCUAGGGCUAACAGCA	12.71319
hsa-miR-3178	AACUGGCCCUCAAAGUCCCGCU	12.26441
hsa-miR-181a	CAAAGUGCUGUUCGUGCAGGUAG	10.09024
hsa-miR-31	AAUGGAUUUUUGGAGCAGG	9.909428
hsa-miR-345	AAGGAGCUUACAAUCUAGCUGGG	9.848006
hsa-miR-663	AAUGACACGAUCACUCCCGUUGA	9.702424
hsa-miR-193a-5p	UAGCUUAUCAGACUGAUGUUGA	9.571058
hsa-miR-1260b	GCAGUCCAUGGGCAUAUACAC	8.908698
hsa-miR-99b	UAACAGUCUACAGCCAUGGUCG	8.887395
hsa-miR-151-3p	UCCUUCAUUCCACCGGAGUCUG	8.517569
hsa-miR-500	CAUUGCACUUGUCUCGGUCUGA	7.33227
hsa-miR-762	CGGGGUUUUGAGGGCGAGAUGA	6.080536

∗When the two arms of precursor were added to produce mature miRNA separately, according to cloning experiments.

**Table 3:** Relationship between miR-375 expression and tumor clinicopathologic features.

Characteristic	Number of cases	miR-375 low level	miR-375 high level	Values
All samples	40	20	20	
Age				
≤60	23	11	12	0.749
>60	17	9	8	
pT status				
T_is_-T_2_	15	3	12	0.744
T_3_-T_4_	25	17	8	
pN status				
N_0_	28	13	15	0.490
N_>0_	12	7	5	
Clinical UICC stage				
Early stage (I-II)	12	2	10	0.006
Advanced stage (III-IV)	28	18	10	
Pathological grade				
G1	14	6	8	0.736
G2-G3	26	14	12	
Tobacco hobby				
No	9	3	6	0.292
Yes	31	17	14	
Alcohol hobby				
No	19	9	10	0.332
Yes	21	11	10	
